# Prevalence of gastrointestinal and blood parasites in horses of Nakhon Si Thammarat province, Thailand

**DOI:** 10.14202/vetworld.2024.2460-2468

**Published:** 2024-11-05

**Authors:** Tanakorn Phetkarl, Punpichaya Fungwithaya, Kittima Lewchalermvong, Narin Sontigun

**Affiliations:** 1 Akkhraratchakumari Veterinary College, Walailak University, Nakhon Si Thammarat 80160, Thailand; 2 One Health Research Center, Walailak University, Nakhon Si Thammarat, 80160, Thailand; 3 Office of Administrative Interdisciplinary Program on Agricultural Technology, School of Agricultural Technology, King Mongkut’s Institute of Technology Ladkrabang, Bangkok 10520, Thailand

**Keywords:** blood parasites, gastrointestinal parasites, horses, southern Thailand

## Abstract

**Background and Aim:**

The global prevalence of gastrointestinal (GI) and blood parasite infections in horses is a significant concern due to their substantial impact on morbidity, mortality, and economic losses in the horse industry. In Thailand, limited research has been conducted on these parasites in horse populations, and data from southern Thailand are lacking. Consequently, this study aimed to estimate the prevalence of GI and blood parasites in horses in Nakhon Si Thammarat province, Thailand.

**Materials and Methods:**

In total, 79 fecal and blood samples were collected from horses across 11 farms in Nakhon Si Thammarat province. The fecal examination was conducted using simple flotation, formalin-ethyl acetate sedimentation, and a modified McMaster technique. Conventional polymerase chain reaction (PCR) was used to identify blood and strongyle parasites. The influence of sex, age, and body condition score on the prevalence of GI parasites was also analyzed.

**Results:**

Six GI parasites were detected: four nematodes (*Oxyuris equi*, *Parascaris equorum*, strongyles, and *Strongyloides westeri*), one trematode (*Gastrodiscus aegyptiacus*), and one protozoan (*Eimeria leuckarti*). The overall prevalence of GI parasites was 74.7%, with single strongyle infections accounting for the highest proportion at 50.6%, followed by co-infections of strongyles and *G. aegyptiacus* at 10.1%. All 11 pooled strongyle samples were positive for cyathostomins and *Strongylus vulgaris* using conventional PCR with specific primers. Sex was significantly associated with the overall prevalence of GI parasites, whereas both sex and age were significant risk factors for infection by strongyle parasites. *Theileria equi* was the only blood parasite species detected in the surveyed horses, with a prevalence of 1.3% (n = 1/79).

**Conclusion:**

This study is the first to estimate the prevalence of GI and blood parasites in horses from Nakhon Si Thammarat province, Thailand. These findings highlight the importance of implementing control measures against GI parasites and are pivotal for developing effective infection prevention strategies.

## Introduction

Horses (*Equus caballus*) play a crucial role in human life, serving as livestock, pets, and companions. They significantly contribute to sports, therapy, and tourism, which are essential to local and national economies. Therefore, maintaining horses in good health and disease control can reduce the owner’s treatment expenses. In Thailand, horses are reared through several methods, including traditional (allowing horses to roam freely in large pastures, a common practice in rural areas), intensive (keeping horses in stables or small enclosures and feeding them a controlled diet of hay, grain, and supplements), and semi-intensive (allowing horses some time in pastures while also providing stable care). Horses grazing on pasture and those exposed to inadequate management practices are at higher risk of gastrointestinal (GI) and blood parasite infections, leading to considerable morbidity and mortality. These infections contribute to economic losses in the horse industry. The prevalent parasites found in horse populations worldwide encompass non-migratory small strongyle (Cyathostominae) and migratory large strongyle (Strongylinae), the ascarid *Parascaris equorum*, and the threadworm *Strongyloides westeri*, predominantly affecting foals and young horses [1–3]. Furthermore, pinworms (*Oxyuris equi*), tapeworms from the Anoplocephalidae family, and botfly larvae (*Gasterophilus* spp.) are commonly encountered in horses. These parasites have been associated with a variety of clinical signs, including weakness, restlessness, unthriftiness, retarded growth, abdominal distension, diarrhea, abdominal pain, and death, particularly in young and immunocompromised horses [4]. In addition, horses can harbor many parasites without exhibiting any clinical signs [5], leading to the spread of parasites to other horses within the herd.

*Babesia caballi*, *Theileria equi*, *Trypanosoma* spp. (e.g., *Trypanosoma vivax*, *Trypanosoma brucei*, and *Trypanosoma evansi*), and *Anaplasma phagocytophilum* are significant blood parasites that affect horses globally [6–8]. These parasites cause diseases known as equine piroplasmosis (EP for *B. caballi* and *T. equi*), equine trypanosomosis, and equine granulocytic anaplasmosis (EGA), respectively, with *Trypanosoma* spp. and *A. phagocytophilum* being zoonotic [9, 10]. EP results in severe symptoms, including fever, anemia, hemoglobinuria, jaundice, anorexia, thrombocytopenia, rapid breathing, rapid heartbeat, hepatomegaly, and splenomegaly, and sometimes death [11], leading to significant economic losses in the horse industry. *T. equi* is generally considered more pathogenic and a more consistent cause of hemoglobinuria and death in equines, whereas *B. caballi* typically causes a more chronic condition characterized by fever and anemia. Among *Trypanosoma* spp., *T. evansi*, the causative agent of surra, affects various hosts with diverse clinical signs. Infected horses, which are the most susceptible species, often suffer from acute forms that frequently lead to death. The clinical signs include fever, weight loss without loss of appetite, bilateral epiphora, anemia, and dependent edema, including in the genital area. Nervous symptoms may appear once the parasite crosses the blood-brain barrier, inevitably leading to death [12]. For *A. phagocytophilum*, the most common symptoms of EGA include fever, depression, loss of appetite, leg swelling, reluctance to move, and gum jaundice.

In Thailand, only a few studies have focused on GI [13] and blood parasites in horses [14, 15], with reports of *B. caballi*, *T. equi*, and *T. evansi* in northern and northeastern regions over the past decade. Given the significant impact of these parasites on morbidity, mortality, and economic losses in the horse industry, as well as the lack of detailed and recent information on these parasites in horses in southern Thailand, understanding their epidemiology is crucial for developing effective prevention and control strategies.

Therefore, this study aimed to estimate the prevalence of GI and blood parasites in horses in Nakhon Si Thammarat province, Thailand.

## Materials and Methods

### Ethical approval

This study was approved by the Institutional Animal Care and Use Committee of Walailak University (Approval number: WU-ACUC-66023).

### Study period and location

Fecal and blood samples of horses were collected from May 2023 to February 2024 in Nakhon Si Thammarat, Thailand. Among the 11 horse farms surveyed, 8 (72.7%) employed a continuous daytime stocking system with nighttime stall housing and used a combination of pasture and hay as the primary forage source, while 3 (27.3%) maintained horses full-time on pasture.

### Sample collection

Based on the Epi Info™ software (version 7.2.2.6) (Centers for Disease Control and Prevention, Atlanta, GA, USA), using a horse population of 198 in Nakhon Si Thammarat [16] with an expected frequency of 90%, a precise error of 5%, and a confidence level of 90%, an estimated sample size of 76 horses was needed to be collected. Overall, 79 fecal and blood samples from 79 horses from 11 farms in Nakhon Si Thammarat province were collected. Fecal samples were collected directly from the rectum of individual horses using polythene hand gloves, placed individually in a ziplock plastic bag, labeled, kept in an ice box, and transported to the parasitology laboratory at Akkhraratchakumari Veterinary College, Walailak University, where samples were stored at 4°C until analysis. For blood collection, approximately 5 mL of blood samples were individually collected from the jugular vein of each horse and immediately transferred into 5-mL vacuum blood tubes containing ethylenediaminetetraacetic acid (EDTA), placed into an ice box, and transported to the laboratory.

During sample collection, sex, age, and body condition score (BCS) were recorded. The age of the horses was grouped into three categories: young (≤4 years), adult (>4–15 years), and old (>15 years) [17]. The BCS was scored on a scale of 0–5 (0-very poor, 1-poor, 2-moderate, 3-good, 4-fat, and 5-very fat), according to Carroll and Huntington [18].

### Fecal examination

Fecal samples were obtained from 79 horses and analyzed using simple flotation [19] and formalin-ethyl acetate sedimentation [20] techniques to detect the presence of GI parasites, that are, helminth eggs and protozoal cysts or oocysts, under a light microscope (Nikon, USA) at 10× and 40× objective lens. Parasite eggs, cysts, and oocysts were identified based on their morphology using the standard identification key of Soulsby [19]. Furthermore, fecal egg counts (FEC) were obtained using a modified McMaster technique with a detection limit of 25 eggs per gram (EPG) of feces [20]. Furthermore, the intensity of infection in the eggs was classified as low (1–500 EPG), medium (501–1000 EPG), or high (>1000 EPG) [19]. As important strongyle nematodes, especially the cyathostomins and *Strongylus* groups, have a similar egg appearance and are difficult to differentiate from each other, positive strongyle samples were selected for molecular studies.

### Strongyle egg collection

Positive strongyle samples from each farm were pooled. For pooled samples, 5 g of each sample from all horses at individual farms was used. Eggs retrieval from fecal samples were performed using the flotation technique. The egg-containing suspension was centrifuged at 1500× *g* for 5 min. The sediment was resuspended in 10 mL of sterile water and centrifuged at 1500× *g* for 5 min, and pellet dissolution was repeated twice. After three rounds of resuspension and centrifugation, the fecal pellet was resuspended in 0.5 mL sterile water, transferred to a microcentrifuge tube, and centrifuged at 5500× *g* for 5 min. The liquid was completely removed by pipetting, and the pellet was used for DNA extraction.

### DNA extraction and polymerase chain reaction (PCR) amplification

Genomic DNA from strongyle eggs from individual farms was extracted using a SimpleWay™ gDNA prep and PCR set I kit (Biofact, South Korea) following the manufacturer’s protocol. Briefly, eggs were first disrupted by adding 100–200 μL of SLB buffer, 5 μL of Proteinase K, and stainless-steel beads into each 2 mL tube and placing it in a TissueLyser LT apparatus (Qiagen, Germany; Cat No./ID: 85600) for 5 min. Subsequently, a tube was placed in a heat block at 99°C for 10 min, allowed to cool for 2 min, centrifuged at 12879× *g* for 1 min, collected the supernatant into a new tube, and stored at −20°C until use.

Genomic DNA was extracted from blood samples using E.Z.N.A.^®^ blood DNA mini kit (Omega Bio-Tek, Norcross, Georgia, USA) according to the manufacturer’s instructions. The concentration of extracted DNA was measured using a Nano-Drop™ One C Microvolume ultraviolet (UV)-visible spectrophotometer (Thermo Fisher Scientific, Massachusetts, USA) and then stored at −20°C until further analysis.

Conventional PCR was used to detect GI and blood parasite DNA. The primers used are listed in Table-1 [21–25]. For GI parasites, PCR reactions contained 6.25 μL PCR master mix III (2×) (Biofact), 2–3 μL DNA template, primers at 0.4 μM for cyathostomins and *Strongylus vulgaris* (Table-1), and nuclease-free water to a final volume of 12.5 μL. The PCR cycle consisted of an initial denaturation step at 95°C for 2 min, followed by 35 cycles of denaturation at 95°C for 20 s, annealing at 55°C for 30 s, extension at 72°C for 30 s, and final extension at 72°C for 5 min.

**Table-1:** Details of primers used for GI and blood parasite detection.

Parasite	Gene	Primer name	Sequences (5´–3´)	Product size (bp)	References
Gastrointestinal parasite					
Cyathostomins	*5.8S-ITS-2 rRNA*	CyathF CyathR	GACTAGCTTCAGCGATGGA AACGYTGTCATACAGGCACT	350–480	[21]
*Strongylus vulgaris*	*ITS-2*	Sv NC2	GTATACATTAAATAGTGTCCCCCATTCTAG GCAAATATCATTAGATTTGATTCTTCCG	186	[22]
Blood parasite					
*Anaplasma*/*Ehrlichia* spp.	*16S rRNA*	EHR16SR EHR16SD	TAGCACTCATCGTTTACAGC GGTACCYACAGAAGAAGTCC	345	[23]
*Babesia*/*Theileria* spp.	*18S rRNA*	Ba/ThF Ba/ThR	CCAATCCTGACACAGGGAGGTAGTGACA CCCCAGAACCCAAAGACTTTGATTTCTCTCAAG	619	[24]
*Trypanosoma* spp.	*ITS1*	ITS1 CF ITS1 BR	CCGGAAGTTCACCGATATTG TGCTGCGTTCTTCAACGAA	250–710	[25]

ITS=Internal transcribed spacer, rRNA=Ribosomal RNA, GI=Gastrointestinal

To detect the presence of blood parasites, the PCR reaction contained 6.25 μL DreamTaq Green Master Mix (2×) (Thermo Scientific, Vilnius, Lithuania), 1–3 μL DNA template (100–200 ng/μL), primers at 0.4 μM for *Anaplasma* spp. and *Babesia*/*Theileria* spp. and 1 μM for *Trypanosoma* spp. (Table-1), and nuclease-free water to a final volume of 12.5 μL. PCR was performed using a Mastercycler Pro S machine (Eppendorf AG, Hamburg, Germany). The PCR cycle consisted of an initial denaturation step at 95°C for 3 min, followed by 35 cycles of denaturation at 95°C for 30 s, annealing at 58°C (for *Trypanosoma* spp.), 62°C (for *Anaplasma* spp.), or 68°C (for *Theileria/Babesia*) for 30 s, extension at 72°C for 1 min, and a final extension at 72°C for 5 min.

For each assay, genomic DNA samples from known GI and blood parasites were used as positive controls, whereas nuclease-free water was used as negative controls. The PCR products were electrophoretically separated on a 1.5% agarose gel in 1× Tris-acetate-EDTA buffer, stained with SERVA DNA Stain G (Serva, Heidelberg, Germany), and visualized under UV light using the ChemiDoc™ Imaging System (Bio-Rad, USA).

### DNA sequencing

The PCR-positive sample was purified using the E.Z.N.A.^®^ cycle pure kit (Omega Bio-Tek) according to the manufacturer’s instructions and subsequently sent to U2Bio Sequencing Service Co., Ltd. (Bangkok, Thailand) for Sanger sequencing using primers used in PCR. The obtained sequence was manually edited and assembled into complete bidirectional consensus sequences using BioEdit software (version 7.2.5) (https://bioedit.software.informer.com/7.2/) [26]. The obtained results were compared with available sequences in GenBank using the Basic Local Alignment Search Tool (BLAST) (https://blast.ncbi.nlm.nih.gov) for species identification and subsequently deposited in GenBank to obtain the accession number.

### Statistical analysis

All collected data were analyzed using IBM SPSS statistics Software version 29.0 (IBM, Armonk, NY, USA). Descriptive statistics were used to determine the prevalence of the parasites, and the Chi-square test (χ^2^) was used to determine any association between the prevalence of GI parasites and age, sex, and BCS. The prevalence of parasites was determined by dividing the number of animals with parasites by the total number of examined animals. In all analyses, p < 0.05 was considered statistically significant.

## Results

### Prevalence of GI parasites using microscopy

The overall prevalence of GI parasites was 74.7% (n = 59/79) (Table-2). Six GI parasites were detected in this study, comprising four nematodes (*O. equi*, *P. equorum*, strongyles, and *S. westeri*), one trematode (*Gastrodiscus aegyptiacus*), and one protozoan (*Eimeria leuckarti*). The prevalence of single, double, and triple GI parasitic infections is presented in Table-2. Of the 59 positive samples, 44 samples (55.7%) were positive for single infection, 13 samples (16.5%) were positive for double infection, and 2 samples (2.5%) were positive for triple infections (Table-2). Among them, a single infection by strongyles (50.6%; n = 40) was found to be the most abundant GI parasite in the study area, followed by co-infection by strongyles and *G. aegyptiacus* (10.1%; n = 8).

**Table-2:** Prevalence of GI parasitic infections among 79 horses.

GI parasites	No. of positive samples	Prevalence (%)
Single infection		
*Eimeria leuckarti*	1	1.3
*Oxyuris equi*	1	1.3
*Gastrodiscus aegyptiacus*	1	1.3
*Parascaris equorum*	1	1.3
Strongyles	40	50.6
Double infection		
Strongyles and *Gastrodiscus aegyptiacus*	7	8.7
Strongyles and *Oxyuris equi*	2	2.5
Strongyles and *Eimeria leuckarti*	1	1.3
Strongyles and *Strongyloides westeri*	1	1.3
Strongyles and *Parascaris equorum*	1	1.3
Triple infection		
*Parascaris equorum*, *Oxyuris equi*, and strongyles	2	2.5
*Parascaris equorum*, strongyles, and *Gastrodiscus aegyptiacus*	1	1.3
Total	59	74.7

GI=Gastrointestinal

### Overall prevalence of GI parasites in relation to risk factors

The overall prevalence of GI parasites was not significantly associated with sex or BCS (p > 0.05), whereas age was significantly correlated with the prevalence of GI parasites (p < 0.05) (Table-3).

**Table-3:** Factors associated with the overall prevalence of GI parasitic infections of horses.

Factor	Category	No. of examined samples	No. of positive (%)	Chi-square	df	p-value
Sex	Male	31	23 (74.2)	0.006	1	0.936
	Female	48	36 (75.0)			
Age	Young	37	32 (86.5)	7.307	2	0.026
	Adult	39	24 (61.5)			
	Old	3	3 (100.0)			
BCS	Poor	1	1 (100.0)	6.317	3	0.097
	Moderate	28	21 (75.0)			
	Good	44	35 (79.5)			
	Fat	6	2 (33.3)			
Total		79	59 (74.7)			

df=Degree of freedom, BCS=Body condition score, GI=Gastrointestinal

### Intensity of GI nematode infection according to FEC

Among the 57 infected horses, the mean FECs for *O. equi*, *P. equorum*, and strongyles were 805 EPG, 615 EPG, and 708.6 EPG, respectively (Table-4). Only one horse showed high *S. westeri* infection levels. The prevalence of low, moderate, and high infection levels of *O. equi* was 40.0%, 40.0%, and 20.0%, respectively. These levels were 60.0%, 20.0%, and 20.0% for *P. equorum*, respectively. In the case of strongyles, low, moderate, and high infection levels were observed in 52.7%, 29.1%, and 18.2% of cases, respectively (Table-4).

**Table-4:** Infection rates, mean fecal egg counts (expressed as eggs per gram, EPG), and intensity of GI nematodes in infected horses (n = 57).

Parasite	No. of infected horses	Infection rate (%)	Mean EPG (Min-Max)	Infection intensity (%)		

				Low (≤500 EPG)	Moderate (501–1000 EPG)	High (>1000 EPG)
*S. westeri*	1	1.8	N/A	0/1 (0.0)	0/1 (0.0)	1/1 (100.0)
*O. equi*	5	8.8	805 (75-2,350)	2/5 (40.0)	2/5 (40.0)	1/5 (20.0)
*P. equorum*	5	8.8	615 (50-1,300)	3/5 (60.0)	1/5 (20.0)	1/5 (20.0)
Strongyle-type eggs	55	96.5	708.6 (25-5,575)	29/55 (52.7)	16/25 (29.1)	10/55 (18.2)

*S. westeri*=*Strongyloides westeri*, *O. equi*=*Oxyuris equi*, *P. equorum*=*Parascaris equorum*, N/A=Not applicable, EPG=Eggs per gram, GI=Gastrointestinal, Min=Minimum, Max=Maximum

### Factors associated with the infection intensity of GI nematode parasites in horses

The analysis of risk factors for the infection intensity of non-strongyle parasites showed no significant associations with sex, age, or BCS (p > 0.05). Conversely, both age and sex were significantly associated with the infection intensity of strongyles (p < 0.05) (Table-5).

**Table-5:** Factors associated with the intensity of infection by GI nematode parasites in horses (n = 79).

Factor	Category	No. of examined samples (%)	Infection intensity (%)				Chi-square	df	p-value

			Negative	Low	Moderate	High			
Non-strongyles (*O. equi*, *S. westeri*, and *P. equorum*)									
Sex	Male	31 (39.2)	24 (77.4)	3 (9.7)	2 (6.5)	2 (6.5)	7.259	3	0.064
	Female	48 (60.8)	46 (95.8)	0 (0.0)	1 (2.1)	1 (2.1)			
Age	Young	37 (46.8)	29 (78.4)	3 (8.1)	3 (8.1)	2 (5.4)	8.157	6	0.227
	Adult	39 (49.4)	38 (97.4)	0 (0.0)	0 (0.0)	1 (2.6)			
	Old	3 (3.8)	3 (100.0)	0 (0.0)	0 (0.0)	0 (0.0)			
BCS	Poor	1 (1.3)	1 (1.3)	0 (0.0)	0 (0.0)	0 (0.0)	5.632	9	0.776
	Moderate	28 (35.4)	27 (96.4)	1 (3.6)	0 (0.0)	0 (0.0)			
	Good	44 (55.7)	36 (81.8)	2 (4.5)	3 (6.8)	3 (6.8)			
	Fat	6 (7.6)	0 (0.0)	0 (0.0)	0 (0.0)	0 (0.0)			
Strongyles									
Sex	Male	31 (39.2)	11 (35.5)	4 (12.9)	10 (32.3)	6 (19.4)	13.752	3	0.003
	Female	48 (60.8)	13 (27.1)	25 (52.1)	6 (12.5)	4 (8.3)			
Age	Young	37 (46.8)	8 (21.6)	11 (29.7)	12 (32.4)	6 (16.2)	13.267	6	0.039
	Adult	39 (49.4)	16 (41.0)	15 (38.5)	4 (10.3)	4 (10.3)			
	Old	3 (3.8)	0 (0.0)	3 (100.0)	0 (0.0)	0 (0.0)			
BCS	Poor	1 (1.3)	0 (0.0)	1 (100.0)	0 (0.0)	0 (0.0)	7.378	9	0.598
	Moderate	28 (35.4)	9 (32.1)	10 (35.7)	6 (21.4)	3 (10.7)			
	Good	44 (55.7)	11 (25.0)	16 (36.4)	10 (22.7)	7 (15.9)			
	Fat	6 (7.6)	4 (66.7)	2 (33.3)	0 (0.0)	0 (0.0)			

*S. westeri*=*Strongyloides westeri*, *O. equi*=*Oxyuris equi*, *P. equorum*=*Parascaris equorum*, df=Degree of freedom, GI=Gastrointestinal, BCS=Body condition score

### Identification of strongyle-type eggs by PCR

**Figure-1:**
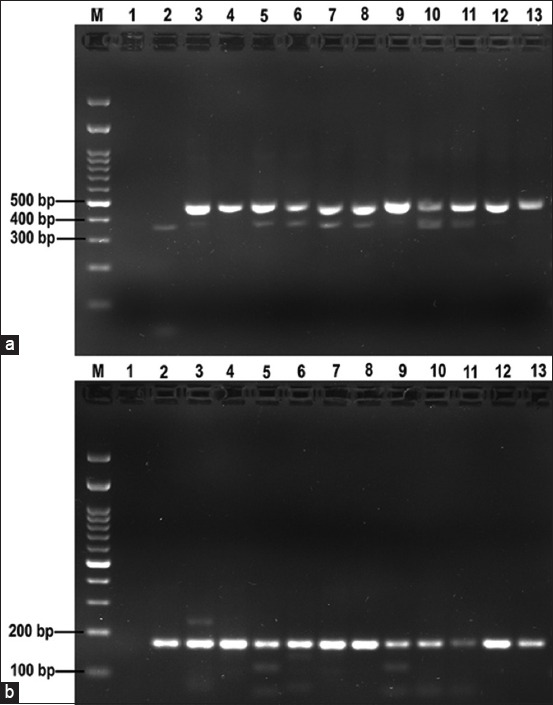
Detection of (a) cyathostomins and (b) *Strongylus vulgaris* in pooled strongyle-type egg samples collected from 11 farms. Lane M, 100-bp DNA ladder; lane 1, negative control; lane 2, positive control; lanes 3–13, individual infected fecal samples.

Eleven pooled strongyle-type egg samples collected from 11 farms were examined by PCR using specific primers specific for cyathostomins and *S. vulgaris*. An expected 350–480 bp fragment of cyathostomins and 186-bp fragment of *S. vulgaris* were detected in all samples (Figure-1).

### The prevalence of blood parasites

Of the 79 blood samples analyzed by PCR, only one sample tested positive using Ba/ThF and Ba/ThR primers targeting the 18S ribosomal RNA of piroplasms (*Babesia*/*Theileria*) (Table-1). *Anaplasma* and *Trypanosoma* DNA were not detected in any samples. BLAST searches identified a positive sample as *T. equi* (MT463613), with 99.38% similarity. Subsequently, the *T. equi* sequence of this study was deposited in GenBank under the accession number PP862533.

## Discussion

This is the first study to estimate the prevalence of GI and blood parasites in horses in Nakhon Si Thammarat province, Thailand.

Globally, the prevalence of GI and blood parasite infections in horses has raised significant concerns due to their effects on morbidity, mortality, and economic losses in the equine industry [1–4, 6–8]. However, only a few studies have investigated these parasites in horse populations in Thailand over the past decade [13–15]. Consequently, there is a scarcity of recent and detailed information regarding GI and blood parasites in horses in Thailand, including southern Thailand.

In this study, the overall prevalence of GI parasites was 74.7%, with strongyles accounting for the highest proportion at 50.6%, followed by coinfection by strongyles and *G. aegyptiacus* at 10.1%. The high prevalence of strongyles was consistent with previous findings in other countries [2, 27–29]. The six GI parasites identified in this study included *E. leuckarti*, *O. equi*, *P. equorum*, strongyles, *S. westeri*, and *G. aegyptiacus*. These findings differ from those of other studies [1, 2, 30, 31] that reported a more diverse range of species. Differences in the identified species and prevalence of GI parasites could be attributed to factors such as sample sizes, sampling collection periods, identification methods, climate, geographical regions, and management systems [32]. The low prevalence of *E. leuckarti* (2.5%), *S. westeri* (1.3%), *O. equi* (3.8%), *P. equorum* (3.8%), and *G. aegyptiacus* (11.4%) observed in this study is consistent with previous studies by Mathewos *et al*. [2], Adeppa *et al*. [33], and Gorji *et al*. [34]. The prevalence of *E. leuckarti* oocysts (2.5%) in this study was found in two adult horses (6 and 14 years old). This finding is inconsistent with the previous reports, which frequently reported *E. leuckarti* oocysts in the feces of equids from many countries with a low prevalence and more commonly in foals rather than adults [34, 35]. In this study, *S. westeri* exhibited the lowest prevalence at 1.3%, and it was identified in a 5-month-old foal. This observation aligns with earlier findings, indicating that *S. westeri* commonly occurs at low prevalence across different regions, including Brazil (0.68%) [29], Switzerland (<1%) [36], and Germany (1.3%) [37], with a notable impact on foals and young horses. The prevalence rate of *P. equorum* (3.8%) documented in our study appears to be relatively lower than rates reported globally by various researchers, such as 10.7% reported by Adeppa *et al*. [33] and 17.0% reported by Elmajdoub *et al*. [38]. Moreover, our study identified *P. equorum* infections in horses aged 3 months–3 years, consistent with the observations of Bucknell *et al*. [30], who noted that this infection predominantly affected young horses under 3 years old, with cases typically confined to animals under 5 years old. The low prevalence of *O. equi* (3.8%) detected in this study is consistent with the previous studies by Mathewos *et al*. [3], Molento *et al*. [29], and Adeppa *et al*. [33] (ranging from 0.75% to 14.1%). This finding may be related to the sampling technique and diagnostic method used; samples were not directly collected from the rectum using the adhesive tape technique, which is recommended for *O. equi* due to the deposition of eggs by gravid females in the perineal region [39]. In our study, prevalence of *G. aegyptiacus* was 11.4%, surpassing previous reports, including rates of 2.9%–10.6% in Ethiopia [2, 40, 41] and 7.1% in India [33]. The variations in the prevalence of *G. aegyptiacus* could be attributed to differences in ecological conditions that influence the development of intermediate snails and the parasite within the study area.

In this study, strongyles were the most frequently detected group of parasites (69.6%). Other studies from various geographical regions have reported even higher prevalence rates [28, 29, 42, 43]. Due to the similar egg appearances of important strongyle nematodes, especially the cyathostomins and *Strongylus* groups, molecular analysis is typically employed for accurate identification. Cyathostomins (small strongyles) are the most prevalent and pathogenic parasites in adult horses, significantly affecting animal health and welfare [44, 45]. Among the large strongyles, *S. vulgaris*, *S. equinus*, and *S. edentatus* were commonly detected, with *S. vulgaris* being the most prevalent and pathogenic species. Therefore, confirmation of the identities of cyathostomins and *S. vulgaris* in fecal samples is urgently required. In this study, both cyathostomins and *S. vulgaris* were detected in all positive strongyle samples (11 pooled samples) by PCR, resulting in a 100% infection rate at the farm level. This finding is consistent with Hinney *et al*. [44], who reported a cyathostomin prevalence close to 100% at the farm level. However, the 100% prevalence of *S. vulgaris* in this study is in contrast with other studies, in which the prevalence was reported to be up to 88% [46, 47].

The analysis of sex, age, and body condition on the overall prevalence of GI parasite infection in the present study indicated that only age was significantly associated with the prevalence (p < 0.05). Specifically, older horses exhibited a higher prevalence of GI parasitic infection compared to young horses (n = 32/37; 86.5%) and adult horses (n = 24/39; 61.5%). This result is in contrast with findings from other studies [2, 3, 31], which reported significant associations between the prevalence of GI parasite infections and factors such as body condition, sex, breed, season, and feed type. For strongyles, the most prevalent parasitic group, infection intensity was significantly correlated with sex and age (p < 0.05) in the horses. This result agrees with the findings of Upjohn *et al*. [27] and Scala *et al*. [48].

In Thailand, few studies on blood parasites in horses have been conducted over the past decade [14, 15], and no studies have been carried out in the southern region. Kamyingkird *et al*. [15] estimated the seroprevalence and molecular detection of *T. equi* and *B. caballi* in horses and mules in Chiang Mai, Thailand. Based on molecular detection, they found a 1.25% *T. equi* infection rate in mules, whereas no *B. caballi* was detected in either horses or mules. In this study, out of 79 horses examined, only one tested positive for *T. equi*, resulting in a prevalence of 1.3% (n = 1/79). This prevalence is lower than that found in other countries, such as 39.9% in Nigeria [7], 78.8% in Mexico [49], and 84.3% in Brazil [50]. Variations in *T. equi* prevalence among horses may be due to differences in geographic areas, sample sizes, intensity of tick infestation, stages of disease at the time of sample collection (acute or chronic), and preventive measures (e.g., treatment of infected animals or tick vector control) [49].

The limited number of samples and collection sites in this study may have influenced the findings related to the prevalence of GI and blood parasites in the examined areas. To enhance the understanding of the prevalence and associated risk factors for GI and blood parasitic infections, future research should involve a larger sample size, a broader range of collection sites, the use of multiple diagnostic techniques for parasite detection, an extended sampling period to account for seasonal variations, and data on farm management practices, drug use, and deworming protocols during farm visits.

## Conclusion

This study represents the first estimation of the prevalence of GI and blood parasites in horses from Nakhon Si Thammarat province, southern Thailand. The GI parasites identified were *E. leuckarti*, *O. equi*, *P. equorum*, strongyles (cyathostomins and *S. vulgaris*), *S. westeri*, and *G. aegyptiacus*, with strongyles being the most prevalent. The statistical analysis revealed that sex was significantly associated with the overall prevalence of GI parasites, while both sex and age were significant risk factors influencing the infection intensity of strongyle parasites. *T. equi* was the only blood parasite species detected in the examined horses. These findings highlight the need for control measures against GI parasites and are essential for creating effective strategies to prevent infections and reduce economic losses in the horse industry.

## Authors’ Contributions

TP: Collected fecal and blood samples, performed the laboratory examination, analyzed the data, interpreted the results, and drafted the manuscript. PF: Performed the laboratory examination and revised the manuscript. KL: Analyzed the data and drafted and revised the manuscript. NS: Designed and supervised the study, performed the laboratory examination, analyzed the data, interpreted the results, and drafted and revised the manuscript. All authors have read and approved the final manuscript.
